# Molecular detection of *Rickettsia* species in ticks collected from the southwestern provinces of the Republic of Korea

**DOI:** 10.1186/s13071-016-1955-x

**Published:** 2017-01-10

**Authors:** Yoontae Noh, Yeong Seon Lee, Heung-Chul Kim, Sung-Tae Chong, Terry A. Klein, Ju Jiang, Allen L. Richards, Hae Kyeong Lee, Su Yeon Kim

**Affiliations:** 1 Division of Zoonoses, National Institute of Health, Centers for Disease Control and Prevention, Cheongju-si, Chungcheongbuk-do 28159 Republic of Korea; 2 5th Medical Detachment, 168th Multifunctional Medical Battalion, 65th Medical Brigade, Unit 15247, Yongsan US Army Garrison, Seoul, APO AP 96205-5247 Republic of Korea; 3 Public Health Command District-Korea, 65th Medical Brigade, Unit 15281, Yongsan US Army Garrison, Seoul, APO AP 96205-5281 Republic of Korea; 4 Viral and Rickettsial Diseases Department, Naval Medical Research Center, Silver Spring, MD 20910 USA

**Keywords:** *Rickettsia*, Spotted fever group rickettsiae, Ixodid ticks, 17 kDa antigen gene, *ompA*

## Abstract

**Background:**

Rickettsiae constitute a group of arthropod-borne, Gram-negative, obligate intracellular bacteria that are the causative agents of diseases ranging from mild to life threatening that impact on medical and veterinary health worldwide.

**Methods:**

A total of 6,484 ticks were collected by tick drag from June-October 2013 in the southwestern provinces of the Republic of Korea (ROK) (Jeollanam, *n* = 3,995; Jeollabuk, *n* = 680; Chungcheongnam, *n* = 1,478; and Chungcheongbuk, *n* = 331). Ticks were sorted into 311 pools according to species, collection site, and stage of development. DNA preparations of tick pools were assayed for rickettsiae by 17 kDa antigen gene and *ompA* nested PCR (nPCR) assays and the resulting amplicons sequenced to determine the identity and prevalence of spotted fever group rickettsiae (SFGR).

**Results:**

*Haemaphysalis longicornis* (4,471; 52 adults, 123 nymphs and 4,296 larvae) were the most commonly collected ticks, followed by *Haemaphysalis flava* (1,582; 28 adults, 263 nymphs and 1,291 larvae), and *Ixodes nipponensis* (431; 25 adults, 5 nymphs and 401 larvae). The minimum field infection rate/100 ticks (assuming 1 positive tick/pool) was 0.93% for the 17 kDa antigen gene and 0.82% for the *ompA* nPCR assays. The partial 17 kDa antigen and *ompA* gene sequences from positive pools of *H. longicornis* were similar to: *Rickettsia* sp. HI550 (99.4–100%), *Rickettsia* sp. FUJ98 (99.3–100%), *Rickettsia* sp. HIR/D91 (99.3–100%), and *R. japonica* (99.7%). One sequence of the partial 17 kDa antigen gene for *H. flava* was similar to *Rickettsia* sp. 17kd-005 (99.7%), while seven sequences of the 17 kDa antigen gene obtained from *I. nipponensis* ticks were similar to *R. monacensis* IrR/Munich (98.7–100%) and *Rickettsia* sp. IRS3 (98.9%).

**Conclusions:**

SFG rickettsiae were detected in three species of ixodid ticks collected in the southwestern provinces of the ROK during 2013. A number of rickettsiae have been recently reported from ticks in Korea, some of which were identified as medically important. Results from this study and previous reports demonstrate the need to conduct longitudinal investigations to identify tick-borne rickettsiae and better understand their geographical distributions and potential impact on medical and veterinary health, in addition to risk communication and development of rickettsial disease prevention strategies.

## Background

*Rickettsia* species are obligate intracellular bacteria in the order Rickettsiales that infect a variety of vertebrate hosts, including humans via arthropod vectors [1]. The genus *Rickettsia* has been classified according to morphological, antigenic, and metabolic characteristics, but now with the availability of genetic information, new approaches to phylogenetic inferences have provided new perspectives on rickettsial classification and evolution. Members of the genus *Rickettsia* are divided into many different phylogenetic groups and this progression will continue with additional phylogeny data. Currently there exists: (i) the spotted fever group *Rickettsia* (SFGR) (e.g. *Rickettsia conorii*, *R. rickettsii* and *R. japonica*, the causative agents of Mediterranean, Rocky Mountain, and Japanese spotted fever, respectively, that are transmitted by ixodid ticks); (ii) the typhus group (TGR) (e.g. *R. typhi*, the causative agent of murine typhus transmitted by fleas, and *R. prowazekii*, the causative agent of epidemic typhus transmitted by the body louse); (iii) the transitional group (TRGR) transmitted by fleas, mites and ticks; (iv) the *R. bellii* group (ticks); (v) the *R. canadensis* group; (vi) the Helvetica group; (vii) the Scapularis group; (viii) the Adalia group; and (ix) the Hydra group [1–4].

Ticks, obligate parasites of vertebrates and found in various natural environments throughout the world, are divided into three families: Ixodidae (hard ticks), Argasidae (soft ticks), and Nuttalliellidae (one species in South Africa). Worldwide, ixodid ticks (e.g. *Haemaphysalis flava*, *H. longicornis*, *Ixodes persulcatus* and *I. nipponensis* in Asia; *I. ricinus* in Europe; *Rhipicephalus sanguineus*, *Dermacentor andersoni*, *D. variabilis*, *Amblyomma americanum* and *Am. maculatum* in America) are the primary vectors/reservoirs of a wide range of rickettsiae of medical and veterinary importance (e.g. *R. japonica*, *R. rickettsii*, *R. conorii*, *R. honei*, *R. sibirica*, *R. slovaca* and *R. monacensis*) that affect birds, wild and domestic animals, and humans in Japan, Mongolia, South Korea, Russia and China [5–11].

SFGR were first reported in Korea based on serological analysis of acute febrile patients [12, 13]. The first case of Japanese spotted fever and isolation of SFGR from a patient in Korea was reported in 2005 [14]. These serological positive sera were assessed by molecular methods based on sequences of the *ompB* gene by nested PCR (nPCR) demonstrated similarities to *R. conorii*, *R. akari*, *R. japonica* and *R. sibirica*.

*Haemaphysalis longicornis* ticks from Chungju Province were positive for *R. japonica* using PCR analysis and sequencing of the *groEL* gene [15]. Moreover, *R. japonica* and *R. monacensis* were detected in *H. longicornis* by nPCR and sequence analysis of the *gltA*, *ompB*, and 17 kDa antigen genes [16, 17]. More recently, *Rickettsia* species have been detected in various arthropods and tick species in Korea that were collected from small mammals, reptiles, and the environment (by tick drag) [10, 18–20].

The purpose of this study was to identify the presence and prevalence of *Rickettsia* species in ticks collected from the southwestern provinces (Jeollanam, Jeollabuk, Chungcheongbuk and Chungcheongnam) of Korea during 2013 to identify and genetically characterize the rickettsiae based on sequence analysis of the partial 17 kDa antigen and *ompA* genes.

## Methods

### Sample collection

A total of 6,484 unengorged ticks (adults, nymphs and larvae) were collected by tick drags when ticks were active during June-October from the southwestern provinces (Jeollanam, Jeollabuk, Chungcheongnam and Chungcheongbuk) of Korea in 2013 as described by Chong et al. [21, 22]. Ticks were identified to species level using morphological keys [23, 24] and placed in 2 ml cryovials according to collection date, species and stage of development (*n* = 6,484; 311 pools of 1–5 adults, 1–25 nymphs, and 1–69 larvae) (Table 1) [25]. Ticks were washed in 70% ethanol, rinsed twice with sterile PBS, and then homogenized in 600 μl of PBS and stored at -70 °C until used for DNA extraction.

**Table 1:** Numbers of pooled ticks collected from the southwestern provinces of Chungcheongnam, Chungcheongbuk, Jeollanam and Jeollabuk in the Republic of Korea

No. of ticks	No. of pools			
	Larvae	Nymphs	Adult males	Adult females
1–5	27	50	19	43
6–9	15	10	0	0
10–19	25	6	0	0
20–29	14	7	0	0
30–39	14	0	0	0
40–49	12	0	0	0
50–59	10	0	0	0
60–69	59	0	0	0
Total	176	73	19	43

### DNA extraction

DNA was extracted from 200 μl of tick suspension using the G-spin total DNA extraction kit (iNtRON, Gyeonggi, Korea) according to the manufacturer’s instructions. DNA was eluted into 50 μl TE buffer and stored at -20 °C until PCR amplification.

### Nested PCR (nPCR) amplification

Direct amplification by nPCR was performed to identify target genes using the partial 17 kDa and *ompA* genes for *Rickettsia* species belonging to the family *Rickettsiaceae*. The *ompA* gene encoded for the SFGR-specific 190 kDa outer membrane protein and the partial 17 kDa antigen gene encoded for the *Rickettsia* genus-specific 17 kDa outer membrane protein.

*Rickettsia* spp. DNA presence was screened using the 17 kDa antigen gene by nPCR as described previously [26]. Briefly, the PCR was performed in a final reaction volume of 20 μl containing 3 μl DNA, 10 pmol of each primer, and the premix reagent (Maxime PCR PreMix kit/i-starTaq^TM^ GH, iNtRON, Gyeonggi, Korea). nPCR was performed in a final reaction volume of 20 μl containing 3 μl of the initial PCR product, 10 pmol of each primer and the premix reagent. Samples positive for the 17 kDa gene target nPCR (appropriate size band identified following agarose gel electrophoresis) were subsequently assessed for the presence of a fragment of *ompA*. The premix reagent, reaction volumes, DNA templates and the amount of primers were the same as those used in the 17 kDa reactions (Table 2).

**Table 2:** Primer sequences and nested PCR conditions for detection of rickettsial target genes from ticks collected from four southwestern provinces of Chungcheongnam, Chungcheongbuk, Jeollanam, and Jeollabuk in the Republic of Korea

Target gene	Primer name	Nucleotide sequence (5'–3')	Product size (bp)	PCR profile (°C/s)				Reference
				Denaturation	Annealing	Extension	Cycles	
17 kDa	Rr17k. 1p	TTTACAAAATTCTAAAAACCAT	539	95/30	57/60	72/120	35	[23]
	Rr17k. 539n	TCAATTCACAACTTGCCATT						
	Rr17k. 90p	GCTCTTGCAACTTCTATGTT	450	95/30	57/60	72/120	35	
	Rr17k. 539n	TCAATTCACAACTTGCCATT						
*ompA*	Rr190k. 71p	TGGCGAATATTTCTCCAAAA	650	95/30	42/35	60/120	35	[23]
	Rr190k. 720n	TGCATTTGTATTACCTATTGT						
	Rr190k. 71p	TGGCGAATATTTCTCCAAAA	532	95/30	48/60	65/120	35	
	Rr190k. 602n	AGTGCAGCATTCGCTCCCCCT						

### Sequencing and phylogenetic analysis

Sequencing of *Rickettsia*-positive nPCR amplicons was conducted by Macrogen Inc. (Daejeon, Korea). The obtained sequences were compared for similarity to sequences deposited in GenBank using BLAST. Gene sequences, excluding the primer regions, were aligned using the multisequence alignment program in Lasergene version 8 (DNASTAR, USA), and phylogenetic analysis performed using MEGA 6 software.

Phylogenetic trees were constructed in CLUSTAL W of the MegAlign Program (DNASTAR, USA) based on the alignment of rickettsial gene sequences obtained following nPCR using the neighbor-joining method and bootstrap analysis (1,000 reiterations) carried out according to the Kimura 2-parameter method. Pairwise alignments were performed with an open-gap penalty of 10 and a gap extension penalty of 0.5. All positions containing alignment gaps and missing data were eliminated during the pairwise sequence comparison (pairwise deletion).

## Results

### Collection of ticks

**Fig. 1:**
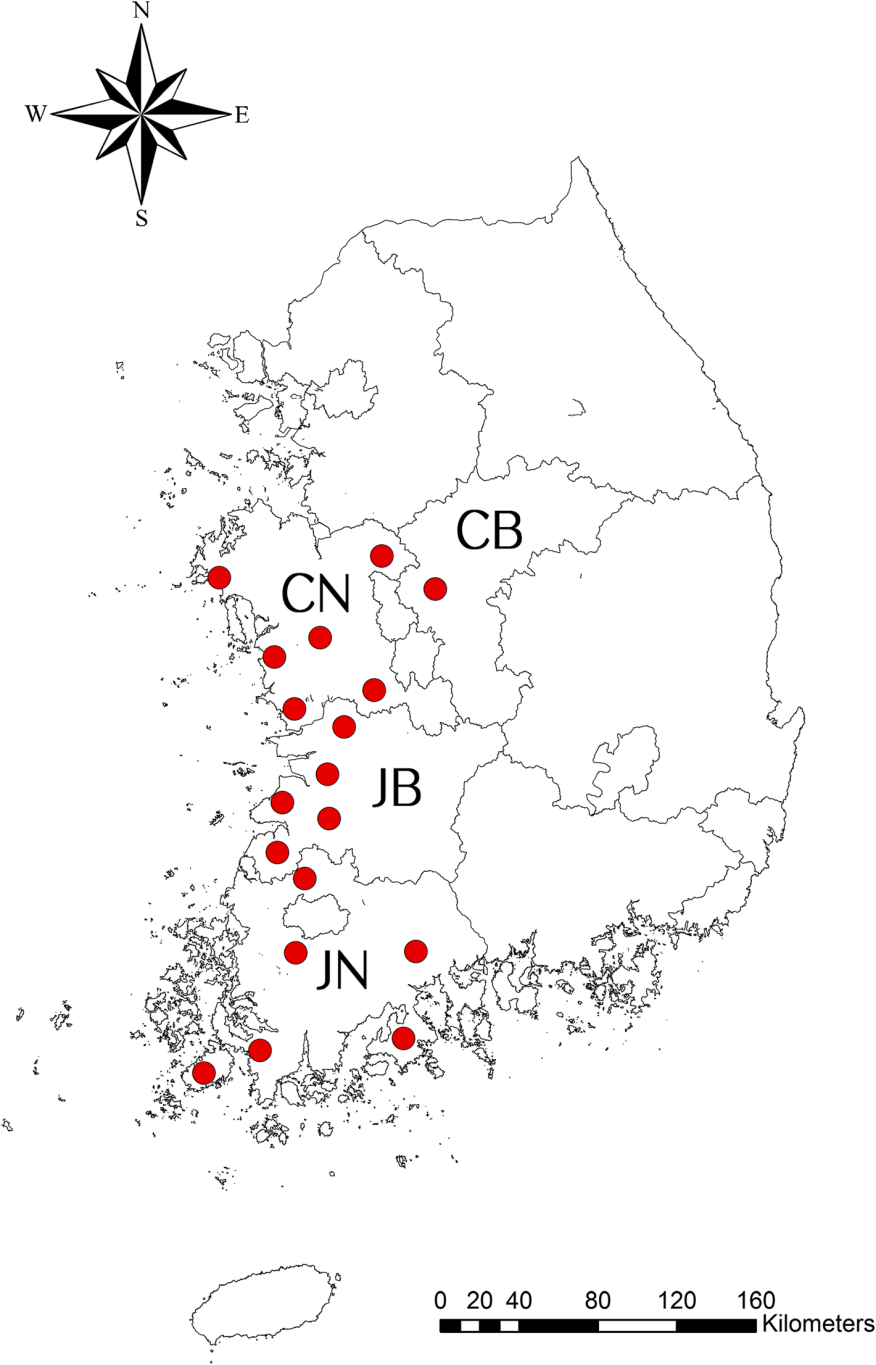
Geographical locations of the tick collection sites in this study. The locations of tick collection sites are marked as *red* closed circles. This map was created using ArcGIS v.10.3.1 software (Environmental Research System Institute, Redland, CA, USA)]. *Abbreviations*: CN, Chungcheongnam Province; CB, Chungcheongbuk Province; JN, Jeollanam Province; JB, Jeollabuk Province

A total of 6,484 ticks belonging to two genera and three species were collected at four southwestern provinces by tick drag (Fig. 1). *Haemaphysalis longicornis* (4,471; 52 adults, 123 nymphs and 4,296 larvae), was the most commonly collected tick, followed by *H. flava* (1,582; 28 adults, 263 nymphs and 1,291 larvae), and *I. nipponensis* (431; 25 adults, 5 nymphs and 401 larvae).

### Detection and prevalence of rickettsial agents

A total of 60/311 (19.30%) pools from Chungcheongnam (3/1,478; 0.20%), Chungcheongbuk (0/331; 0%), Jeollanam (53/3,995; 1.33%) and Jeollabuk (4/680; 0.59%) provinces, respectively, were positive for *Rickettsia* spp. using the 17 kDa antigen gene nPCR assay (Table 3). A total of 51/168 (30.36%) and 46/168 (27.38%) of *H. longicornis* pools were positive for *Rickettsia* using the 17 kDa antigen and *ompA* genes, respectively. Only 1/108 (0.93%) and 0/108 (0%) of *H. flava* were positive for *Rickettsia* spp. using the partial 17 kDa and *ompA* genes, respectively, while 7/35 (20.00%) pools of *I. nipponensis* were positive for *R. monacensis*.

**Table 3:** SFGR minimum field infection rates (MFIR) for ticks collected from four southwestern provinces of Chungcheongnam, Chungcheongbuk, Jeollanam and Jeollabuk in the Republic of Korea during 2013 by province

Province	Species	Stage	No. of ticks (No. of tested pools)	nPCR positive no. of *Rickettsia* sp. (MFIR, %)^a^	
				17 kDa	*ompA*
Chungcheongnam	*H. longicornis*	Larva^b^	533 (22)	1 (0.19)	1 (0.19)
		Nymph^c^	16 (9)	0	0
		Adult (male)^d^	1 (1)	0	0
		Adult (female)^d^	22 (10)	0	0
	*H. flava*	Larva	475 (17)	0	0
		Nymph	30 (8)	0	0
		Adult (male)	5 (2)	0	0
		Adult (female)	3 (2)	0	0
	*I. nipponensis*	Larva	379 (13)	0	0
		Nymph	3 (2)	0	0
		Adult (male)	5 (3)	1 (20.00)	1 (20.00)
		Adult (female)	6 (2)	1 (16.66)	1 (16.66)
		Subtotal	1,478 (91)	3 (0.20)	3 (0.20)
Chungcheongbuk	*H. longicornis*	Larva	147 (6)	0	0
		Nymph	2 (2)	0	0
		Adult (female)	3 (2)	0	0
	*H. flava*	Larva	176 (5)	0	0
		Nymph	1 (1)	0	0
	*I. nipponensis*	Larva	2 (1)	0	0
		Subtotal	331 (17)	0	0
Jeollanam	*H. longicornis*	Larva	3,314 (70)	34 (1.03)	31 (0.94)
		Nymph	103 (18)	7 (6.79)	6 (5.82)
		Adult (male)	6 (4)	4 (66.66)	4 (66.66)
		Adult (female)	20 (11)	2 (10.00)	2 (10.00)
	*H. flava*	Larva	401 (19)	1 (0.25)	0
		Nymph	105 (18)	0	0
		Adult (male)	4 (3)	0	0
		Adult (female)	11 (8)	0	0
	*I. nipponensis*	Larva	18 (2)	1 (5.55)	0
		Adult (male)	5 (3)	2 (40.00)	2 (40.00)
		Adult (female)	8 (5)	2 (25.00)	2 (25.00)
		Subtotal	3,995 (161)	53 (1.33)	47 (1.18)
Jeollabuk	*H. longicornis*	Larva	302 (11)	2 (0.66)	1 (0.33)
		Nymph	2 (2)	1 (50.00)	1 (50.00)
	*H. flava*	Larva	239 (9)	0	0
		Nymph	127 (11)	0	0
		Adult (male)	2 (2)	0	0
		Adult (female)	3 (3)	0	0
	*I. nipponensis*	Larva	2 (1)	1 (50.00)	1 (50.00)
		Nymph	2 (2)	0	0
		Adult (male)	1 (1)	0	0
		Subtotal	680 (42)	4 (0.59)	3 (0.44)
		Total	6,484 (311)	60 (0.93)	53 (0.82)

^a^MFIR (minimum field infection rate/100 ticks) = no. of positive pools/no. of examined ticks in pools × 100, by species and stage of development

^b^1–69 larvae/pool

^c^1–25 nymphs/pool

^d^1–5 adults/pool

The overall minimum field infection rates (MFIR) of *Rickettsia*-positive pools (assuming 1 positive tick/pool) were 0.93% (60/6,484) for the 17 kDa antigen gene and 0.82% (53/6,484) for the *ompA* gene targets (Table 4) [25]. The overall MFIR for all three species ranged from 0–0.88% for larvae, 0–6.50% for nymphs, and 0–57.1% for adults (Table 4). There were no significant differences (Chi-square test, *P* = 0.98) observed between the positive rates of the partial 17 kDa and *ompA* genes.

**Table 4:** SFGR minimum field infection rates (MFIR) for ticks collected from four southwestern provinces of Chungcheongnam, Chungcheongbuk, Jeollanam and Jeollabuk in the Republic of Korea during 2013 by species of tick using the partial 17 kDa and ompA genes by nPCR

Collected ticks	Developmental stage	No. of ticks/(no. of tested pools)	nPCR positive no. of *Rickettsia* sp. (MFIR)^a^	
			17 kDa	*ompA*
*H. longicornis*	Larva^b^	4,296 (109)	38 (0.88)	33 (0.77)
	Nymph^c^	123 (31)	8 (6.50)	7 (5.70)
	Adult (male)^d^	7 (5)	4 (57.10)	4 (57.10)
	Adult (female)^d^	45 (23)	2 (4.40)	2 (4.40)
	Subtotal	4,471 (168)	52 (1.16)	46 (1.03)
*H. flava*	Larva	1,291 (50)	0	0
	Nymph	263 (38)	1 (0.38)	0
	Adult (male)	11 (7)	0	0
	Adult (female)	17 (13)	0	0
	Subtotal	1,582 (108)	1 (0.06)	0
*I. nipponensis*	Larva	401 (17)	1 (0.25)	1 (0.25)
	Nymph	5 (4)	0	0
	Adult (male)	11 (7)	3 (27.2)	3 (27.2)
	Adult (female)	14 (7)	3 (21.4)	3 (21.4)
	Subtotals	431 (35)	7 (1.62)	7 (1.62)
	Totals	6,484 (311)	60 (0.93)	53 (0.82)

^a^MFIR (minimum field infection rate/100 ticks) = No. of positive pools/No. of examined ticks in pools × 100, by species and stage of development

^b^1–69 larvae/pool

^c^1–25 nymphs/pool

^d^1–5 adults/pool

### Sequencing and phylogenetic analysis

The partial 17 kDa antigen gene and *ompA* nPCR amplicons were sequenced and aligned with other rickettsial genes deposited in the GenBank database to identify known sequences with a high degree of similarity using ClustalW [26]. The sequencing electropherograms of all positive pools were confirmed as single peaks, indicating each pool represented a single *Rickettsia* species.

**Fig. 2:**
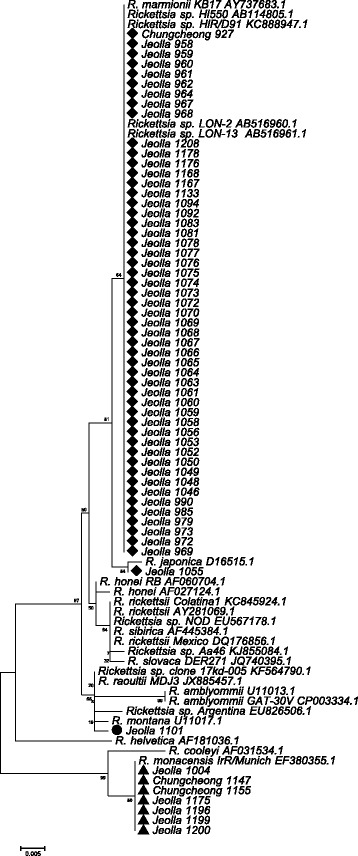
Phylogenetic tree based on 342 bp of the 17 kDa outer membrane protein gene of *Rickettsia* species

**Fig. 3:**
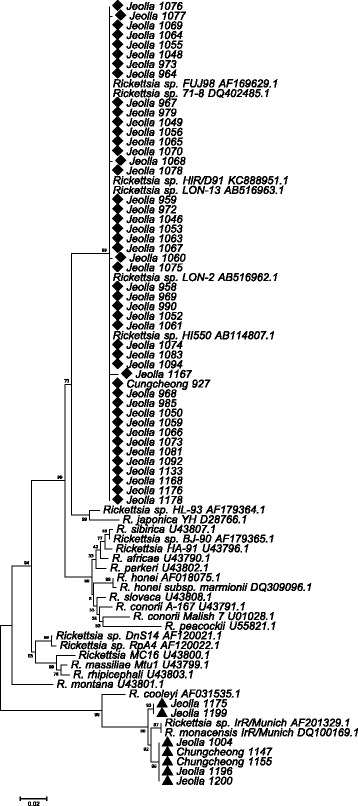
Phylogenetic tree based on 443 bp of *ompA* gene of *Rickettsia* species

The amplicon sequences of the partial 17 kDa gene obtained from *H. longicornis* demonstrated 99.4–100% similarity to previously reported molecular sequences from *H. longicornis* that phylogenetically clustered with *Rickettsia* sp. HIR/D91, *Rickettsia* sp. 71-8, *Rickettsia* sp. HI550 and *Rickettsia* sp. LON-2, LON-13. Similarly, *ompA* sequences of positive pools of *H. longicornis* demonstrated 99.3–100% similarity to sequences of rickettsiae from *H. longicornis* previously reported as *Rickettsia* sp. HIR/D91, *Rickettsia* sp. LON-2, LON-13, *Rickettsia* sp. HI550 and *Rickettsia* sp. FUJ98, that are similar, but distinct from *R. japonica.* Phylogenetic analysis showed a close relationship between rickettsial isolates from *H. longicornis* from the southwestern provinces of Korea and rickettsial isolates from *H. longicornis* from other Asian countries (Figs. 2 and 3).

The amplicon sequence of the partial 17 kDa gene for one positive pool of *H. flava* demonstrated 99.7% similarity to previously reported *Rickettsia* sp*.* 17-kDa-005 that clustered with *R. montanensis* and *R. raoultii* (Fig. 2). The amplicon sequences of the partial 17 kDa gene and *ompA* genes from seven pools of *I. nipponensis* demonstrated 100% and 98.7–98.9% similarity, respectively, to previously reported *R. monacensis* IrR/Munich (Figs. 2 and 3).

## Discussion

Tick-borne rickettsiae are obligate intracellular bacteria belonging to the genus *Rickettsia*, many of which are of medical importance [27–29]. Clinically, tick-borne rickettsioses present with mild to life threatening signs and symptoms that include: an eschar (not always indicated) that is present 1–2 days prior to the onset of headache and fever (39.5–40.0 °C), and a characteristic rash 1–2 days after the onset of fever that can last for 2–3 weeks. Tick-borne infections are often reported as non-specific febrile diseases due to the lack of specific clinical signs and symptoms and diagnostic assays effective early in the process of disease [27–29]. In the USA, there were a total of 3,649 cases of rickettsioses reported between 1997–2002 and more than 1,500 cases reported annually since 2005 (www.cdc.gov/rmsf/stats/index.html). This increase in reporting may be due to the fact that rickettsioses are becoming more widely recognized [1, 29]. In addition to disease producing tick-borne rickettsiae, many rickettsiae (e.g. *R. bellii*, *R. canadensis*, *R. asiatica*, *R. hoogstraalii*, *R. montanensis*, *R. rhipicephali* and *R. tamurae*) have not been identified as pathogens and therefore are often referred to as non-pathogenic or of unknown pathogenicity. To further complicate matters, with the discovery of numerous new *Rickettsia* spp. in ticks using molecular tools, their role as causative agents of diseases of medical and veterinary importance has not been established, in part owing to lack of diagnostic tools for pathogen detection, rather than for antibodies [29].

With increased interest in tick-borne diseases, surveillance of ticks from reptiles, mammals, birds, and vegetation has led to the identification of known and yet to be described pathogens belonging to genera of *Ehrlichia*, *Anaplasma*, *Bartonella*, *Borrelia*, *Babesia* and *Rickettsia*, in addition to viruses [10, 18–20, 30–36]. *Rickettsia akari*, a mite-borne pathogen isolated from a rodent, was first reported in Korea in 1957 [37, 38]. Later, acute febrile patients tested positive by serological tests for *R. japonica* in 2004, 2005 and 2006 [9, 12, 13]. Recently, various gene targets from rickettsial pathogens were identified in various ixodid tick species, including *H. longicornis*, *H. flava*, *I. nipponensis* and *I. persulcatus* [15–17].

*Haemaphysalis longicornis*, *H. flava* and *I. nipponensis* are commonly collected throughout Korea, while *H. phasiana*, *A. testudinarium*, *I. pomerantzevi*, *I. persulcatus* and *I. ovatus* have a limited geographical/habitat distribution and are collected much less frequently [22, 38, 39]. Tick-borne disease surveillance usually includes the detection of pools of ticks, as it is costly and untimely to assay for multiple agents within individual ticks [25]. However, it is important to assay ticks from specific habitats (e.g. forests and grasses/herbaceous vegetation) and hosts over their geographical range to determine the potential association with man and domestic animals, as well as the distribution of associated pathogens [8, 10, 16, 17, 19, 20, 31, 39].

The conserved 17 kDa antigen gene was used in this study to screen for the presence of rickettsiae in tick pools. Subsequently, the 17 kDa *Rickettsia*-positive pools (*n* = 60) were assessed for the presence of *ompA* (also by nPCR). All but seven of the 60 *Rickettsia*-positive pools were positive for the more variable *ompA* gene. A previous report also showed that *ompA* genes were not detected in several different rickettsial genotypes [26].

Results of the partial 17 kDa antigen and *ompA* gene sequences obtained from *H. longicornis* pools showed that the rickettsial agents detected were closely related to *Rickettsia* sp. HIR/D91, 71-8 identified in Korea, *Rickettsia* sp. HI550 and LON-2, LON-13 identified in Japan, and *Rickettsia* sp. FuJ98 identified in China. Only one *Rickettsia*-positive *H. flava* pool sequenced demonstrated a high similarity to *Rickettsia* sp. 17-kDa-005 identified in China, while all seven *Rickettsia*-positive pools of *I. nipponensis* were similar to *R. monacensis* and *Rickettsia* sp. IrR/Munich identified in Europe. *Rickettsia monacensis*, a known human pathogen, was first isolated from *I. ricinus* collected from an English garden in Germany in 1998 [1]. While *R. monacensis* was generally observed only in *I. ricinus* mainly from southern and eastern Europe [40], it has been detected in *I. nipponensis* collected from rodents captured in Korea (Jeollanam Province in 2006 and Gyeonggi and Gangwon provinces in 2008) [10, 17].

*Haemaphysalis longicornis* is commonly collected from grasses and herbaceous vegetation, while *H. flava* is more commonly associated with forest habitats and *I. nipponensis* is collected similarly from both habitats throughout the ROK [22]. *Haemaphysalis longicornis* is commonly found in grassy areas that expose civilians and military populations to tick bites and associated pathogens, which not only include *Rickettsia* spp., but other bacteria and viruses of medical and veterinary importance [32–35, 41]. *Rickettsia monacensis* has been detected in *I. nipponensis*, which are more frequently reported in tick bites [42–45]. While *H. longicornis*, the primary vector of the severe fever with thrombocytopenia syndrome (SFTS) virus, has not been frequently reported to bite humans, with 36, 51, and 78 cases of SFTS infections among civilians in the ROK reported from 2013–2015, respectively, indicates that most bites go unreported with the potential for the transmission of rickettsiae to both civilian and military populations.

Additional analysis of gene sequences of *Rickettsia* spp. will allow for their specific identification and the development of species-specific PCR assays and determination of their medical and veterinary importance. SFGR infections are not reportable events in the ROK and some cases are likely included as scrub typhus since the symptoms, including fever, eschar and rash, are similar. Analysis of eschar tissue by PCR would allow for the detection and identification of *Rickettsia* spp. and scrub typhus strains among patients with similar disease presentation [46]. The identification of the SFGR diseases and scrub typhus is essential to determine tick- and mite-borne disease risks and develop appropriate disease prevention strategies. Additional investigations to determine the identification of rickettsiae associated with each of the tick species using single tick analysis and the geographical/habitat distribution of each of the tick species and associated pathogens are needed to identify disease risks to both civilian and military populations in the ROK.

## Conclusion

Rickettsial pathogens pose a potential health threat to military and civilian communities in the ROK. *Ixodes nipponensis* has been shown to be infected with *R. monacensis*, a human pathogen, and *H. longicornis* and *H. flava* have been shown to be infected with SFGR of unknown pathogenicity. More intense and longitudinal surveillance of ticks and their hosts in the ROK is needed to determine their geographical and habitat distributions, and the geographical distribution and prevalence of their associated pathogens. The characterization of SFGR is essential to identify agents of tick-borne human diseases and their relative pathogenicity. Lastly, the detection and identification data of the rickettsiae reported herein will provide for the development of species-specific diagnostic assays that are essential for rapid detection of SFGR in the vectors, vertebrate hosts and patients.

## Abbreviations

17 kDa: *Rickettsia*-specific outer membrane antigen gene
CB: Chungcheongbuk Province
CN: Chungcheongnam Province
JB: Jeollabuk Province
JN: Jeollanam Province
MFIR: minimum field infection rate per 100 ticks
nPCR: nested PCR
*ompA*: spotted fever group-specific 190 kDa outer membrane protein A gene
ROK: Republic of Korea
